# Real-Time Air-Writing Recognition for Arabic Letters Using Deep Learning

**DOI:** 10.3390/s24186098

**Published:** 2024-09-20

**Authors:** Aseel Qedear, Aldanh AlMatrafy, Athary Al-Sowat, Abrar Saigh, Asmaa Alayed

**Affiliations:** 1 Department of Computer Science and Artificial Intelligence, College of Computing, Umm Al-Qura University, Makkah 21955, Saudi Arabiaabrarsaigh1@gmail.com (A.S.); 2 Department of Software Engineering, College of Computing, Umm Al-Qura University, Makkah 21955, Saudi Arabia

**Keywords:** deep learning, Arabic air-writing recognition, mid-air, Arabic alphabet, hand gestures, fingertips, writing, Arabic language

## Abstract

Learning to write the Arabic alphabet is crucial for Arab children’s cognitive development, enhancing their memory and retention skills. However, the lack of Arabic language educational applications may hamper the effectiveness of their learning experience. To bridge this gap, SamAbjd was developed, an interactive web application that leverages deep learning techniques, including air-writing recognition, to teach Arabic letters. SamAbjd was tailored to user needs through extensive surveys conducted with mothers and teachers, and a comprehensive literature review was performed to identify effective teaching methods and models. The development process involved gathering data from three publicly available datasets, culminating in a collection of 31,349 annotated images of handwritten Arabic letters. To enhance the dataset’s quality, data preprocessing techniques were applied, such as image denoising, grayscale conversion, and data augmentation. Two models were experimented with using a convolution neural network (CNN) and Visual Geometry Group (VGG16) to evaluate their effectiveness in recognizing air-written Arabic characters. Among the CNN models tested, the standout performer was a seven-layer model without dropout, which achieved a high testing accuracy of 96.40%. This model also demonstrated impressive precision and F1-score, both around 96.44% and 96.43%, respectively, indicating successful fitting without overfitting. The web application, built using Flask and PyCharm, offers a robust and user-friendly interface. By incorporating deep learning techniques and user feedback, the web application meets educational needs effectively.

## 1. Introduction

Writing is a fundamental skill for children, enabling them to convey their emotions, ideas, and thoughts effectively. Developing strong writing skills at a young age lays a solid foundation for personal development and academic achievement. Specifically, robust Arabic writing skills provide children with a deep understanding of a language that is gaining global attention [1,2]. Arabic is considered one of the most challenging languages, ranking second in difficulty in 2022 [3]. This underscores our responsibility as Arabic-native speakers to use modern technologies to facilitate language learning and proficiency.

One advanced technique to enhance writing skills is air-writing, also known as skywriting or finger spelling [4]. This method involves using hand or finger movements to write in the air, eliminating the need for physical objects. Air-writing can be used by individuals of all ages.

Recently, air-writing recognition technology, which detects hand or body movements to understand intended gestures, has seen significant advancements. This technology offers numerous benefits across various domains. In accessibility, it helps individuals with physical disabilities, such as those with muscular dystrophy, spinal cord injuries, or cerebral palsy, by providing an alternative means of communication and text input [5]. In education [6], it supports interactive and engaging learning experiences. Additionally, workers in noisy environments can use this technology to interact without relying on physical buttons or touch-based interfaces.

Integrating air-writing recognition technology opens new avenues for effective language learning and writing skill development, particularly for the Arabic language. There are multiple compelling reasons that led us to select this field and, specifically, this idea. Primarily, our choice is driven by the significance of transforming education and is strongly anchored in the Vision 2030 of the Kingdom of Saudi Arabia, which explicitly promotes the widespread adoption of modern teaching methodologies [7]. SamAbjd can be seen as a pioneering effort to utilize air-writing recognition in the education sector.

Moreover, the lack of artificial intelligence [8,9,10] applications in teaching the Arabic language, compared to many other languages, motivated us to enhance language education for children through the development of an air-writing recognition system for the alphabet. Recognizing the significant role of technology in education, SamAbjd aims to leverage advanced technologies, such as artificial intelligence, to offer a unique and interactive approach to learning Arabic.

By using air-writing recognition technology, SamAbjd strives to capture children’s attention and make the learning process enjoyable and exciting. The ability to write in the air and see their movements transformed into a recognizable Arabic alphabet fosters a sense of empowerment and wonder among children, encouraging them to actively participate in their own learning journey.

The paper is structured as follows: Section 2 reviews the relevant literature. Section 3 covers the methodology, beginning with a discussion of gathering and preprocessing the dataset, followed by an overview of the model selection and training process. The methodology section then describes the development of the backend for the air-writing component and concludes with details on the implementation of the frontend user interface for the air-writing system. Results are discussed in Section 4. Section 5 concludes the paper and discusses the future work.

## 2. Related Work

Researchers have proposed various approaches and techniques to recognize air-written characters, utilizing technologies such as depth cameras, fingertip detection, tracking algorithms, and deep learning [11] models. This section will explore several recent research papers in the field of air-writing recognition, highlighting their methodologies and contributions to improving the accuracy and performance of these systems. Itaguchi et al. [12] highlighted the importance of air-writing in kanji and its influence on cognitive processing and motor actions. The study demonstrated that air-writing had varying impacts depending on the task, with visualizing finger movements aiding character identification and specific finger movements affecting counting performance. Air-writing proved beneficial in enhancing task performance, while unrelated finger movements had a negative effect. According to the reviewed literature, air-writing recognition can be divided into four categories based on acquisition devices: sensor-based recognition, radar-based recognition, device-based recognition, and vision-based recognition.

### 2.1. Air-Writing Recognition with Sensor-Based Recognition

In 2013, Xin Zhang et al. [13] developed a finger-writing system that allows users to write characters in the air using the Kinect sensor without the need for an additional handheld device. The system used the Microsoft Kinect sensor to capture depth and color information, creating a finger-writing interface. A depth-skin background was introduced in a mixture model (DSB-MM) for accurate hand segmentation and employed a state-of-the-art handwriting character recognition method. A modified quadratic discriminant function (MQDF) character classifier was used to recognize 6763 Chinese characters, 26 English letters (uppercase and lowercase), and 10 digits. Experiments showed real-time recognition rates exceeding 90%. In 2015, Wang et al. [14] tackled the challenge of recognizing handwritten Chinese characters in air-writing. A discriminative three-level classifier was proposed using learning vector quantization (LVQ) for discriminative prototypes and adaptive discriminative locality alignment (ADLA) to distinguish similar characters. Tested on the IAHCC-UCAS2014 dataset with a Leap Motion controller, this method showed higher recognition accuracy and lower computational cost compared to a basic classifier, offering insights and suggesting future improvements for in-air handwritten Chinese character recognition. In 2019, Mohammadi Shahram et al. [15] tackled the problem of recognizing Persian digits and numbers in air-writing using the Kinect sensor. The method involves extracting a feature vector from the trajectory and employing an analytical classifier for character recognition. The experiments demonstrated a 98% recognition rate for Persian digits and numbers, highlighting the system’s practical usability.

#### 2.1.1. Air-Writing with Radar-Based Recognition

Radar technology is a valuable tool for sensor-based recognition in air-writing. In 2019, Arsalan et al. [16] addressed air-writing recognition by detecting linguistic characters or words written in free space through hand gestures. A two-stage approach was proposed using millimeter-wave radars. The extraction stage captured and processed hand motion trajectories to detect and localize the hand marker. In the recognition stage, the long short-term memory (LSTM) used variants with a connectionist temporal classification (CTC) loss function and a deep convolutional neural network (DCNN) for character classification. The convolutional LSTM with CTC (ConvLSTM-CTC) approach achieved a classification accuracy of 98.33%, comparable to the DCNN’s performance. In 2022, Ahmed et al. [17] explored recognizing mid-air-writing gestures as a promising application for human–computer interfaces. A multistream convolutional neural network (MS-CNN) approach was proposed using a frequency-modulated continuous wave (FMCW) radar. This method involved a three-stream CNN network with range-time, Doppler-time, and angle-time spectrograms as inputs fused for recognition. A dataset of air-written digits from zero to nine was used and collected from twelve volunteers, and the MS-CNN system achieved 95% accuracy, outperforming traditional CNN architectures. The authors also made the radar data available for further research and suggested real-time application exploration and larger datasets for training and testing.

#### 2.1.2. Air-Writing with Devices

Devices play a crucial role in capturing and analyzing hand gestures in air-writing recognition. Various devices, including handheld devices, mobile phones with accelerometers, smart bands, and biometric smart pens, facilitate the recognition of air-written characters and words by collecting hand movement data. In 2011, Muzaffar Bashir et al. [18] introduced a biometric smart pen for personal authentication via air handwriting. Using dynamic time warping (DTW) for time-series data classification, the recognition rates achieved higher than 99.9% for PIN words and characters, with a 2 s response time for a population of 40 users. The same year, Sandip et al. [19] developed the PhonePoint Pen, which used a mobile phone’s accelerometer to capture hand gestures as geometric strokes, translating them into English characters with an average accuracy of 91.9%. In 2016, Mingyu Chen et al. [20] studied air-writing variability across devices, using optical tracking and inertial measurements to collect 6-DOF hand motion data. Hidden Markov models (HMMs) achieved high accuracy, with a word error rate of 0.8% for words and 1.9% for letters. In 2020, Yanay et al. [21] proposed two methods for recognizing air-written letters using smart bands. The user-dependent method with K-nearest neighbors (KNN) and DTW achieved 89.2% accuracy, while the user-independent method with CNN achieved 83.2% accuracy (95.6% with auto-correction).

### 2.2. Air-Writing with Vision-Based Recognition

Air-writing recognition systems often use vision-based technology, leveraging depth-sensing cameras or regular cameras. These systems have gained significant attention due to the widespread availability of devices with built-in cameras, such as tablets and smartphones.

In 2013, Faisal Baig et al. [22] introduced a real-time video-based system for writing English text in the air using a mobile phone’s front camera. This approach tracks the red-colored index finger, extracts its coordinates, and plots them. The system achieved a 92.08% character recognition accuracy but struggled with color sensitivity issues. In 2016, Robiul et al. [23] used the Microsoft Kinect depth camera to capture hand motions for recognizing English capital letters (ECAs). They employed the DTW algorithm, achieving an average accuracy of 96.3%. Future improvements involve collecting additional datasets. Prasun Roy et al. [24], in 2018, presented a CNN framework for recognizing handwritten numerals in air-writing gestures using a video camera. This method, using a fixed-color marker, achieved high recognition accuracy across multiple languages: 97.7% for English, 95.4% for Bengali, and 93.7% for Devanagari. In 2018, Sohom et al. [25] proposed a webcam-based air-writing recognition system. The method used was Faster R-CNN for hand detection and a novel fingertip detection algorithm, achieving 96.11% character recognition accuracy. Shahinur Alam et al. [26], in the year 2020, developed an air-writing identification system using the Intel RealSense SR300 depth camera. By normalizing the trajectory and using CNN and LSTM for recognition, they achieved 99.32% accuracy. Al Abir et al. [27] tackled the challenge of recognizing air-written characters using deep learning techniques and interpolation. The issue of variable signal duration in air-writing data was addressed by proposing interpolation to ensure consistent signals. Using seven publicly available air-writing datasets, the researchers developed a two-dimensional deep convolutional neural network (2D-CNN) model. Their experiments showed that this model outperformed state-of-the-art methods in both user-dependent and user-independent scenarios. The user-dependent model achieved 100% accuracy for digits, while the user-independent model showed an accuracy gain of 3.55% for all classes combined, with specific gains of 0.52%, 1.62%, and 2.24% for digit, lowercase, and uppercase datasets, respectively. In 2021, Chaur-Heh Hsieh et al. [28] explored air-writing recognition for smart TV control using deep CNNs and a single webcam. The system achieved over 99% accuracy for digit and directional symbol recognition. Watanabe Taiki et al. [29], in 2023, employed a hybrid CNN and BiLSTM framework to recognize air-written characters using fingertip positions captured by a webcam. An accuracy of 99.3% was achieved for alphabets and 99.5% for digits. Khalid M. O. Nahar et al. [30] focused on recognizing air-written Arabic letters using a hybrid model that combines feature extraction, deep learning, and optical character recognition (OCR) techniques. Using CNNs like VGG16, their system achieved 88.8% accuracy. Two notable reviews include Zabulis et al. [31], who, in 2020, discussed vision-based hand gesture recognition for human–robot interaction in museums, and in 2021, Liu et al. [32] explored gesture recognition for human–robot collaborative manufacturing.

To conclude, some of the papers faced challenges in finding suitable datasets for air-writing recognition, which led them to collect their own dataset for the study. In contrast, other papers were able to rely on publicly available datasets. While some papers utilized self-collected datasets, others relied on publicly available datasets or created their own to address the lack of data in the field. Additionally, it is worth noting that only one paper specifically addressed the recognition of air-written Arabic letters, highlighting a potential gap in research focusing on Arabic alphabets in the context of air-writing recognition. It is worth mentioning that all of the papers focused solely on utilizing air-writing techniques with models without exploring practical applications. In contrast, our project aims to go beyond the theoretical aspects and apply these techniques in a practical format. Through the development of an application, our goal is to bridge the gap between research and real-world usage, offering a tangible solution that can be utilized by individuals seeking to recognize Arabic alphabets through air-writing.

## 3. Methodology

**Figure 1:**
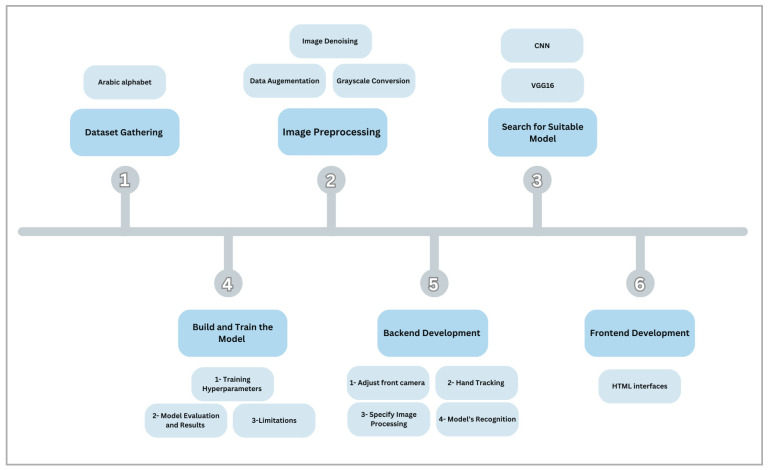
Overview of the methodological steps.

In this study, the researchers collected 31,349 handwritten Arabic letters from three different public sources to create a diverse and representative dataset. They prepared this dataset for analysis by cleaning up the images to remove noise, converting them to grayscale, and enhancing them through techniques like rotation, zooming, and shifting. CNN and the VGG16 model are known for their accuracy in recognizing images. Additionally, the researchers developed a real-time system for recognizing air-written Arabic letters using Python 3.9.13, Flask 3.0.3, OpenCV 4.9.0, and MediaPipe 0.10.10 for the backend. They also created a user-friendly web application for the frontend, designed to make learning intuitive for young children. The methodological steps are illustrated in Figure 1.

### 3.1. Data Gathering

The dataset was carefully constructed by gathering handwritten Arabic letters from three publicly available datasets. The process involved assembling a significant collection of handwritten Arabic letters from these sources. The resulting dataset comprises an impressive total of 31,349 meticulously annotated images, encompassing both black-and-white and colored variations.

The first dataset, available in [33], is a comprehensive collection specifically designed to support research in Arabic handwritten character recognition. This dataset comprises 33,600 handwritten Arabic characters contributed by 60 participants aged between 19 and 40 years, with 90% being right-handed. Each participant was assigned the task of writing each Arabic character, from ‘alef’ ‘ أ’ to ‘yeh’ ‘ ي ‘, ten times on two separate forms. The dataset is further divided into a training set and a test set, with the training set containing 26,880 characters and the test set comprising 6720 characters. Notably, all images in this dataset are presented in black and white. To enhance our own dataset, we utilized only the training set from this initial dataset.

The second dataset, sourced from [34], comprises 16,800 handwritten Arabic characters. The dataset is split into a training set containing 13,440 characters (480 images per class) and a test set with 3360 characters (120 images per class). The images in this data set are red, green, and blue (RGB) images. We incorporated image files from both the training and test sets from this dataset.

The third dataset [35] contains characters and numbers, and the characters appear in various positions—at the beginning, middle, and end of words, as well as in isolation (not within a word). Our focus has been on recognizing the isolated character forms, so we only gather these data.

**Figure 2:**
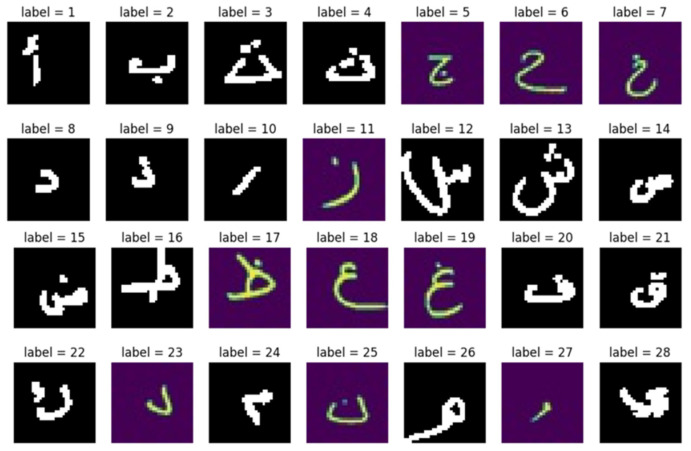
Handwritten Arabic letters dataset.

To facilitate the labeling process, we consolidated three datasets into a single file and renamed them using the following structure: “Label_Letter_numberOfImageForTheLetter” (e.g., “1_alef_1” and “2_beh_1”). Additionally, a comma-separated value (CSV) file was created with a single column containing labels numbered from 1 to 28, representing the images in the dataset file. Each label was repeated based on the number of images associated with that particular letter. Finally, the dataset file and CSV file were merged into one file, which was then uploaded to Google Drive for storage and accessibility. Figure 2 presents sample images for each letter in the dataset.

### 3.2. Dataset Splitting

The final dataset was divided into training, testing, and validation sets. The training set comprised 90% of the total data, while the remaining 10% was allocated for testing. To create the validation set, data augmentation techniques were applied to the test set. This approach enhances the model’s ability to generalize and handle diverse data, a crucial aspect for successful character recognition in real-world applications.

### 3.3. Data Preprocessing

**Figure 3:**
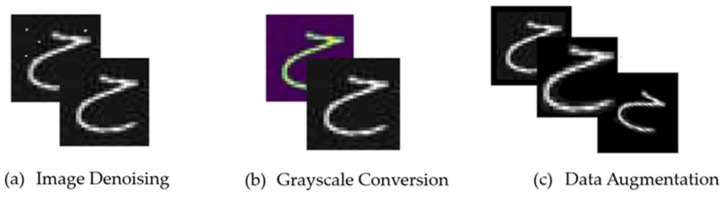
Data preprocessing techniques applied to the dataset.

Data preprocessing is a crucial step in deep learning and computer vision projects. Its primary objective is to transform raw data into a suitable format for training models and improving their performance. In this section, we will provide a detailed explanation of the four data preprocessing techniques that were applied to our dataset: image resizing, denoising, grayscale conversion, and data augmentation. These techniques enhance the dataset’s quality and usability by eliminating noise, inconsistencies, and irrelevant information. As a result, they contribute to more accurate predictions and improved model performance; see Figure 3.

#### 3.3.1. Image Denoising

The first step in the preprocessing phase involved image denoising, which is the process of reducing or removing unwanted noise from an image [36]. The goal was to effectively reduce the noise while preserving the important details and features of the image. The focus was on denoising the images using the non-local means denoising method [37]. This advanced technique preserves the edges and details of the images by averaging the pixel values within small local neighborhoods. The weights for averaging are determined based on the similarity between these neighborhoods, allowing effective noise reduction while keeping important image features intact.

#### 3.3.2. Grayscale Conversion

Another preprocessing technique involved converting the images into grayscale. To determine if an image was already in grayscale, a function for converting to grayscale was implemented. If an image was found to be in color, it was converted to grayscale using the ‘L’ mode. The modified grayscale image was then saved, overwriting the original data.

#### 3.3.3. Data Augmentation

Data augmentation has been applied to generate wide-ranging data variations, aiming to improve the model’s prediction accuracy. Taking into account that the dataset comprises handwritten Arabic letters, it is crucial to exercise caution when selecting suitable data augmentation techniques that do not alter the integrity of the letter structure.

Several common augmentation techniques have been employed for the handwritten letters dataset. Rotation is among these techniques, where the images are randomly rotated within a typical range of 10 degrees. By applying rotation, the model becomes more capable of handling variations in letter orientation, enabling accurate recognition regardless of the rotation angle. This technique introduces slight orientation variations without distorting the fundamental structure of the letters.

By employing zooming, either the scale or size of the images is randomly adjusted. In our case, the zooming range was set to 0.1, meaning the images of handwritten Arabic letters could be magnified or reduced in size by up to 10% of their original dimensions. This random scaling simulates diversities in the letter sizes that can occur during the writing process. Zooming enables the model to become more adaptable to different letter sizes and improves its ability to recognize and classify letters accurately. Additionally, width shifting has been utilized as another augmentation technique. Applying width shifting, the images are horizontally translated by up to 10% of their original width. Consequently, letters can appear slightly displaced toward the left or the right within the image. The intention behind implementing this technique is to simulate differences that can occur during the handwriting process, such as slight changes in the horizontal positioning of the letters.

Finally, the dataset benefits from the implementation of the height-shifting technique, where small random shifts, typically around 10% of the image height, are introduced in the vertical position of the letter images. This enhances the model’s ability to handle variations that can occur during the writing process, such as subtle changes in the vertical alignment of the letters.

### 3.4. Searching for Suitable Neural Network Algorithms

The selection of appropriate algorithms is crucial in computer vision, as it directly impacts the effectiveness of solving specific problems. For the image classification of handwritten Arabic alphabets and numerals, the researchers prioritized precision and accuracy, leading them to choose two highly suitable deep learning models: CNN [38] and the VGG16 architecture [39].

This decision is well supported by the previous literature, as CNN and VGG16 have consistently emerged as successful models for real-time recognition tasks. Researchers have demonstrated significant success in classifying and identifying images of written alphabets using deep learning models like CNNs, which have proven highly effective in image classification [30,40]. These models are designed to learn spatial hierarchies of features, capturing essential characteristics in the early layers and progressively learning more complex patterns in deeper layers. This hierarchical feature extraction is particularly beneficial for the Arabic letters dataset, enabling the models to effectively represent the unique visual properties of Arabic script [41]. Consequently, CNN and VGG16 are exceptionally well suited for recognizing the nuanced visual patterns in Arabic letter images.

#### 3.4.1. The CNN Model

**Figure 4:**
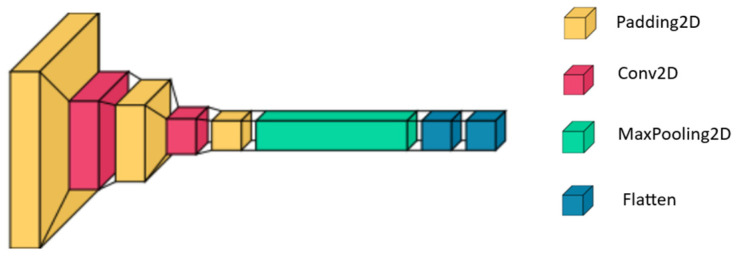
The architecture of the CNN model, inspired by [30].

CNNs [42], as shown in Figure 4, are feed-forward neural networks that learn feature engineering through filter optimization. CNNs are widely used in image classification and offer several advantages over traditional algorithms. They require less preprocessing than other image classification algorithms because they learn to optimize filters using automated learning.

A CNN’s architecture consists of three layers: an input layer, a hidden layer, and an output layer. The hidden layers may include one or more convolutional layers. Similar to how a neuron in the visual cortex responds to a specific stimulus, convolutional layers convolve the input and pass the result to the next layer. Each convolutional neuron processes data exclusively for its receptive field, which is a subset of the previous layer.

The receptive field of a neuron in a CNN is the region from which it receives input. Because of the repeated convolution operation that considers the value of a pixel and its surrounding pixels, each neuron in a convolutional layer receives input from a larger area of the input than previous layers. Alternatives to the standard convolutional layer include atrous or dilated convolution, which increases the receptive field size without increasing the number of parameters.

#### 3.4.2. The VGG16 Model

**Figure 5:**
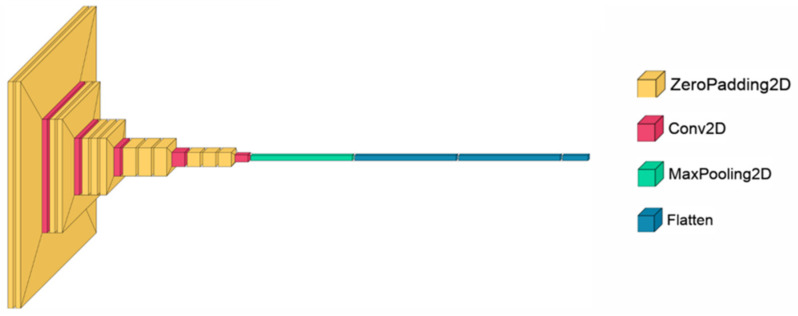
The architecture of the VGG16 model, inspired by [30].

The VGG-16 model [43], shown in Figure 5, also known as VGG16Net, is considered one of the CNN architectures proposed by the VGG for object detection and classification. It is known by its depth, consisting of 16 layers, including 13 convolutional layers and 3 fully connected layers. VGG-16 is deeper compared to other CNN architectures and can capture more complex visual patterns. Its depth, however, makes it more computationally expensive and prone to overfitting. Despite this, VGG-16 is widely used for image-related tasks due to its strong performance.

### 3.5. Build and Train the Model

The core aim of our model is to accurately recognize air-written Arabic letters and effectively distinguish between them with the highest level of performance. In order to achieve optimal results, a comprehensive series of experiments were conducted to evaluate various CNN models with different types and layers, which are CNNs with 7 layers and 9 layers and VGG16 with and without a fully connected layer.

#### 3.5.1. Training Hyperparameters

A series of adjustments were conducted, as shown in Table 1, to the training parameters, specifically targeting the epoch, batch size, and learning rate. In the first set of changes, different epochs were experimented with, trying out values of 20, 25, and 50 to observe their impact on the training process. Simultaneously, the batch size was also modified, exploring the effects of 400, 512, and 515 as batch size values. Additionally, we fine-tuned the learning rate, setting it to 0.001 and 0.02 to optimize the convergence of our model.

Moreover, to further explore the effects of different activation functions on the model’s performance, changes were made to the activation functions used in the convolutional layers. Specifically, the activation functions Scaled Exponential Linear Unit (SELU), Rectified Linear Unit (ReLU), and Stochastic Gradient Descent (SGD) allowed us to evaluate their impact on the model’s ability to learn and generalize.

By combining these tunings, including changes in the number of convolutional layers and activation functions, we aimed to comprehensively analyze the impact of these parameters on the model’s convergence, accuracy, and overall performance.

#### 3.5.2. Model Evaluation and Results

##### Evaluation Metrics

In the field of deep learning, the accurate and precise evaluation of model performance is critical. Accuracy, precision, and the F1-score [44,45] were utilized to measure the model’s performance in training and prediction. They were produced using a confusion matrix, which is a tabular representation made up of four main components:

- True positive (TP): the number of cases accurately classified as positive by the model.
- False positive (FP): cases the model wrongly categorized as positive despite being in the negative class.
- True negative (TN): the number of cases accurately classified as negative by the model.
- False negative (FN): cases the model mistakenly categorized as negative despite being in the positive class.

For a given dataset of size *n*, these metrics are measured as follows [46]:

(1) $$Accuracy=\frac{\left(TP+TN\right)}{\left(TP+FP+TN+FN\right)}$$

(2) $$Precision=\frac{\left(TP\right)}{\left(TP+FP\right)}$$

(3) $$F1-Score=2\times \frac{\left(Precision\times Recall\right)}{\left(Precision+Recall\right)}$$

By using these metrics, the researchers acquired a thorough insight of the performance of the CNN models and VGG16. Accuracy is an overall measure of correctness, precision is concerned with the quality of positive predictions, and the F1-score combines precision and recall, providing a balanced evaluation. These metrics allow us to evaluate the model’s capacity to accurately classify images, reduce false positives, and strike a balance between precision and recall.

##### Experiments and Results

The researchers performed a thorough comparison of two models, the CNNs with different layer configurations and VGG16 with and without a fully connected layer. The results of this analysis are shown in Table 2.

The result of the analysis of various models was insightful when looking at their performance on the given metrics. For most CNN models, adding layers generally resulted in higher training and validation accuracy percentages. Dropout layers were introduced in the CNN models in order to avoid overfitting and to achieve better generalization. Nevertheless, this addition to the models may not outperform models without dropout in terms of accuracy.

On the VGG16 models, the actual inclusion of the fully connected layer had a more subtle effect on the training performance. Without the fully connected layer, the accuracy percentages dropped to between 72.75% and 80.66%, signifying that this layer is necessary for accurate feature extraction and classification.

When comparing models, it can be observed that the CNN models clearly scored better in accuracy with respect to the VGG16 model, along with higher training, testing, validation accuracies and higher precision and F1-score percentages, which means the CNN models are better at identifying the positive instances and maintaining a good balance between true positives and false negatives.

**Figure 6:**
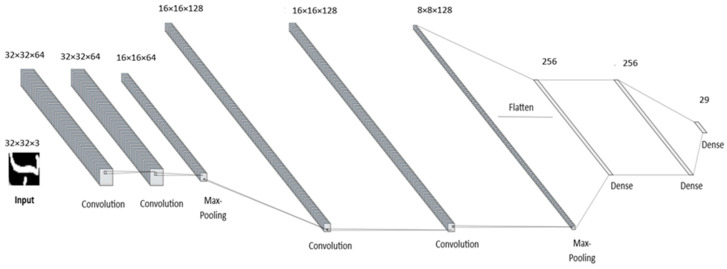
The architecture of the CNN model used in this study, inspired by [30].

The CNN model with seven layers and without dropout, as seen in Figure 6, performed better than any other CNN model. This is shown by its impressive training accuracy of 95.92%, validation accuracy of 94.70%, and testing accuracy of 96.40%, reflecting strong learning and generalization powers. Its training loss was low, amounting to only 12.46%, while validation loss was 16.46% accurate, leaving no room for overfitting. The seven-layer CNN model scored higher than the rest; this can be noted from its precision (96.43%) and F1-score (96.44%).

**Figure 7:**
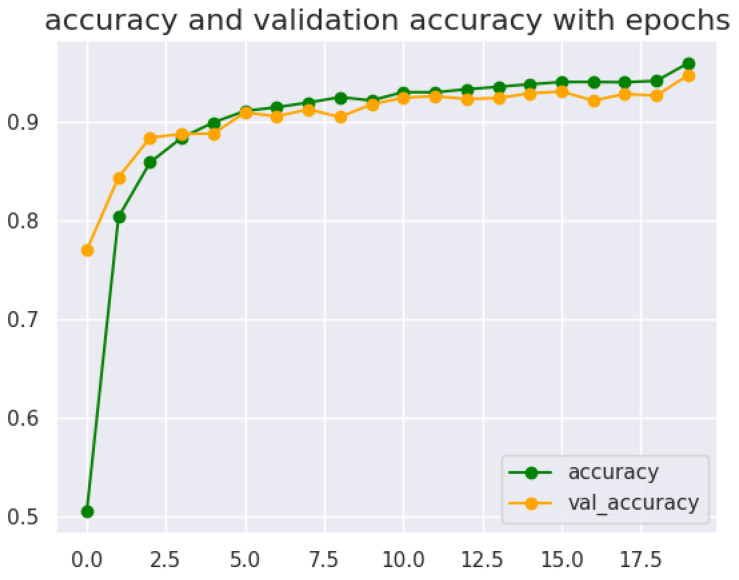
Training and validation accuracy of CNN 7 layers with no dropout model.

The decision to select the CNN model with seven layers and no dropout, despite not achieving the highest accuracy, was due to the balance between training accuracy, testing accuracy, and validation accuracy, as seen in Figure 7. The model can leverage its feature extraction capabilities to capture the small details of the Arabic alphabet in real-time input. By avoiding overfitting or underfitting, the model can adapt to the variations in the air-written alphabet and generalize well to unseen examples. This allows for accurate classification of the Arabic alphabet based on the real-time input from the webcam.

**Figure 8:**
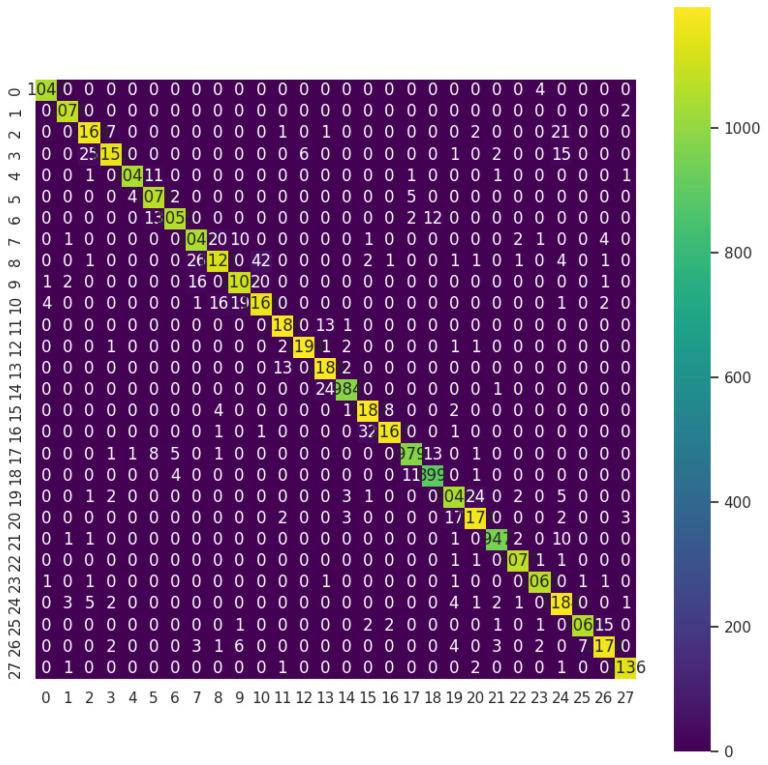
Confusion matrix of CNN 7 layers with no dropout model.

An analysis of Figure 8, depicting the confusion matrix for a CNN with seven layers and no dropout, offers invaluable insights into the model’s proficiency in classifying air-written letters. These letters are labeled from 0 to 27, representing the 28 letters of the Arabic alphabet. Through examination, the matrix reveals the model’s accuracy, highlighting areas of precision and potential misclassification across the full spectrum of Arabic letters. By aligning anticipated letter labels with columns and actual letter labels with rows, the matrix unveils patterns of confusion, aiding in the identification of specific challenges faced by the model in accurately recognizing each letter. Specifically, the confusion matrix reveals that the model tends to confuse labels 7 and 8, representing the Arabic letters “د” (dal) and “ذ” (thal), respectively, due to their similar shapes.

The resemblance between these two letters’ shapes likely contributes to the model’s difficulty in distinguishing between them accurately. Similarly, the model also shows confusion between labels 2 and 3, which correspond to the Arabic letters “ت” (teh) and “ث” (theh), respectively. These letters have similar shapes, differing only in the number of dots, which may explain the model’s tendency to misclassify them.

#### 3.5.3. Limitations

During the training for the seven-layer CNN model, we encountered the following key limitations:

Certain letters are challenging for the model to recognize effectively because they have similar structures. For example, the model may misclassify the letter ‘أ’ (alef) as ‘ع’ (ain) because of the presence of the ‘ء’ in some images from the dataset, making it appear like the start of the ‘ع’ letter. To face this, we compare the model’s confidence score for the ‘أ’ letter to a specific threshold value. If the confidence score reaches or surpasses this threshold, the model will identify the input image as the ‘أ’ letter. There is still room for growth in this regard.

The researchers also implemented a confidence score threshold condition for letters without dots to ensure they are recognized consistently, and the model will not mix them up with the ones that have a similar structure.

### 3.6. Backend Development

#### 3.6.1. Air-Writing Recognition in Real-Time

The proposed method involves multiple stages for building the air-writing components, specifically (1) air-writing tools, (2) building the air-writing component, and (3) air-writing samples.

##### Air-Writing Tools

To implement the air-writing component effectively, a combination of software and hardware tools is utilized to provide reliable development and testing environments.

Google Colab 3.10.12 [47] is used as an online collaborative platform by Google, designed for writing and running Python code. Access to powerful hardware is offered, and seamless integration with Google Drive [48] allows for efficient storage and sharing of work. This tool is particularly useful for experimenting with machine learning models and sharing results with researchers. Additionally, PyCharm [49], developed by JetBrains, is employed as a powerful integrated development environment (IDE) tailored specifically for Python development. Comprehensive coding, debugging, and testing functionalities are provided. PyCharm also facilitates collaboration among developers, making it an essential tool for complex Python projects.

The hardware setup for the air-writing component involves the use of multiple laptops, each chosen for its high-performance specifications to ensure smooth and efficient operation. The Lenovo Legion 5 15ACH6H features an AMD Ryzen 7 5800 H processor with Radeon Graphics, a 3.20 GHz CPU, and 16.0 GB of RAM. The Vostro 14 5401 is powered by an Intel(R) Core(TM) i7 processor with a base speed of 1.30 GHz (boost up to 1.50 GHz) and 16.0 GB of RAM. The IdeaPad 5 Pro 16IHU6 is equipped with an 11th Gen Intel(R) Core(TM) i7-11370H processor running at 3.30 GHz and 16.0 GB of RAM. Lastly, the Alienware m15 r5 features an AMD Ryzen 5800 CPU and 16.0 GB of RAM. Together, these laptops create a robust environment for developing, testing, and refining the air-writing component, ensuring optimal performance and collaborative efficiency.

##### Building Air-Writing Component

The web application was developed using Python 3.9.13, selected for its compatibility with the essential libraries used in the project, such as Flask 3.0.3, OpenCV 4.9.0, and MediaPipe 0.10.10. MediaPipe, in particular, requires specific Python versions for optimal performance and compatibility, with Python 3.9.13 providing the best stability and feature support for these integrations.

The development occurred in two phases. The first phase focused on hand movement tracking and image capture, enabling functionalities such as writing in the air and recognizing various gestures. During this phase, the camera was opened, and the hand’s movements were continuously tracked. These movements were interpreted to support interactions for writing and recognizing different gestures, such as using the index finger for writing, two fingers for pausing, and raising the full hand to start recognition. Once the images were captured, image processing was applied to prepare them for input into the model.

**Figure 9:**
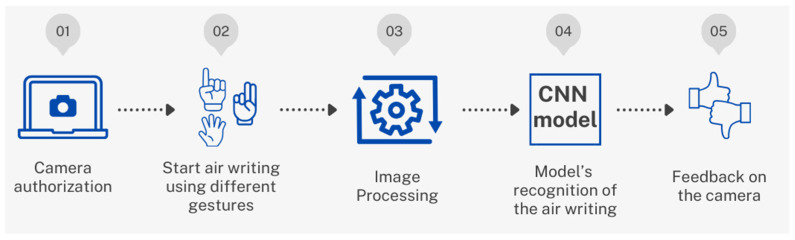
The flow for the backend system.

The second phase centered on real-time recognition of air-writing. This recognition was achieved by using a pre-trained model capable of understanding air-written letters. Upon the recognition of the writing, feedback on the correctness of the writing was provided, ensuring an enjoyable and enhanced learning experience. This process is illustrated in Figure 9.

#### 3.6.2. Adjust Front Camera

The camera functionality is central to the operation of the web application, enabling real-time interaction with the user. The webcam is accessed by the web application using OpenCV, a library specifically designed for computer vision tasks. OpenCV allows for the capture and processing of video frames in real time, which are essential for subsequent analysis and interaction within the web application.

#### 3.6.3. Hand Tracking

Hand tracking is applied in the air-writing component, implemented by using MediaPipe, a cross-platform framework from Google for building machine learning pipelines that support multiple input modalities. As part of the developed air-writing component, MediaPipe enabled a real-time hand-tracking process that relied on a pre-trained machine learning model to predict the location of specific hand landmarks in each video frame.

The process of hand tracking starts by examining each video frame to specify if the hand is apparent. For the visible hand, the component identifies specific points on the hand, known as landmarks. Landmarks serve as reference points for identifying hand gestures. Based on these analyses, the backend generates appropriate responses from one of the three responses: enabling drawing in the air, pausing air-writing temporarily, or starting the recognition process.

Within the air-writing component, specific gestures are used to control the way of the writing and start the recognition process. Identifying these gestures using MediaPipe involved analyzing the positions of specific landmarks on hand. A set of predefined hand landmarks, such as the tips of the fingers and the base of the palm, supported by MediaPipe, can be used to track the hands’ poses and movements.

**Figure 10:**
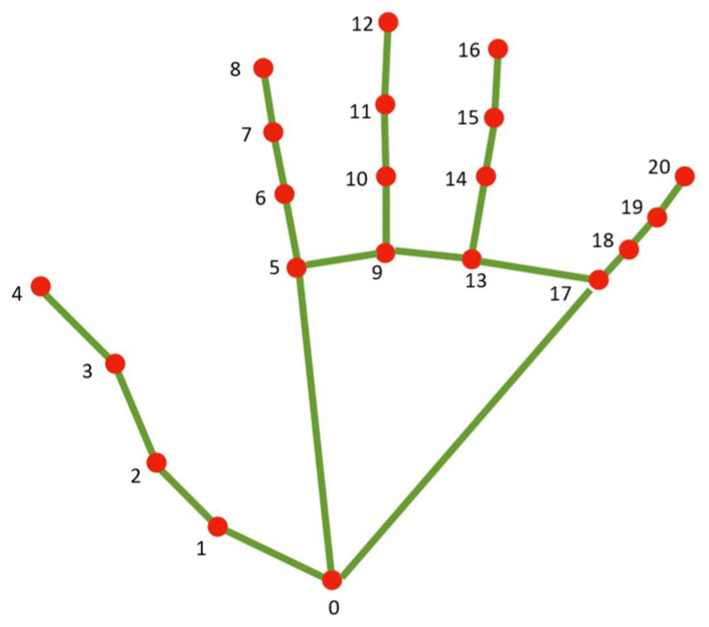
Hand landmarks for fingertips, adopted from [50].

The core interaction begins by raising the index finger—the index (fingertip 8). As shown in Figure 10, the component can track the movement of the index fingertip landmark. As the index finger moves in the air, the trajectory of the fingertip is recorded. Through continuous tracking and analysis of the index fingertip’s position, the path of the finger’s movement can be reconstructed and represented as a line on the camera, simulating writing in the air.

To add diacritical marks (such as dots) common in Arabic letters or to temporarily pause the writing, the user raises two fingers, the index (fingertip 8) and middle (fingertip 12) fingers, as illustrated in Figure 10. The system detects this two-finger gesture by analyzing the relative positions of the fingertips and lower finger joints.

Finally, to signal the completion of a written letter and start the recognition process, the user fully raises their hand, as depicted in Figure 10. This prompts the system to analyze the captured finger movements and interpret the written content.

**Figure 11:**
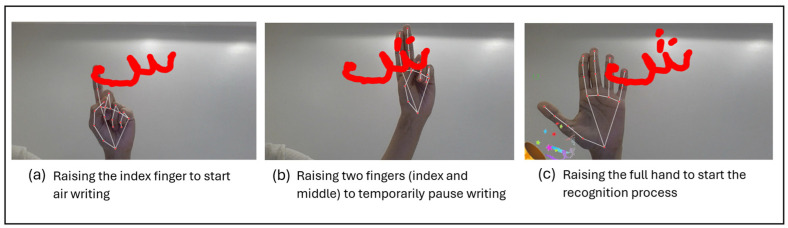
Steps of writing a letter in the air.

Figure 11 illustrates the steps for writing the letter ‘shen’ (ش) in the air. Begin by raising your index finger. Next, use two fingers to determine the positioning of each dot (.). Finally, employ your full hand to enable letter recognition and provide appropriate feedback.

The algorithm used in the air-writing component to identify hand gestures is shown in Algorithm 1.
**Algorithm 1.** Algorithm for detecting hand gesturesFUNCTION are_all_fingers_up(hand_landmarks): DEFINE fingertip_indices as a list of indices for the fingertip landmarks DEFINE lower_joint_indices as a list of indices for the lower joint landmarks INITIALIZE finger_status as a list of False values, one for each finger # Check index finger separately GET index_finger_tip and index_finger_lower_joint landmarks from hand_landmarks IF index_finger_tip.y < index_finger_lower_joint.y: SET finger_status [1] to True # Index finger is up # Check the remaining fingers FOR each finger (starting from the middle finger): GET the fingertip and lower joint landmarks IF fingertip.y < lower_joint.y: SET the corresponding finger_status to True # Finger is up # Check if all fingers are up IF finger_status [1] AND finger_status [2] AND finger_status [3] AND finger_status [4] are all True: RETURN True, finger_status ELSE: RETURN False, finger_status

#### 3.6.4. Image Processing

Our study investigated the processing of camera input and canvas images to prepare data for a pre-trained deep learning model. During the writing process, the camera input and the canvas image are combined and synchronized. This synchronization ensures that any content present in the camera is accurately reflected in the canvas, and vice versa. As a result, to prepare the input for the pre-trained model, it is necessary to work with a copy of the original canvas.

The procedures for processing the canvas images involved the following:

##### Handling White Writing

**Figure 12:**
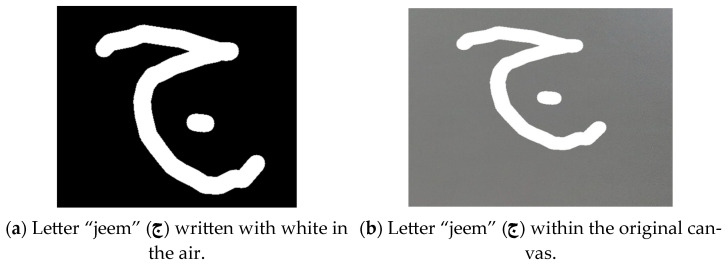
Creating a bounding box around the letter.

As depicted in Figure 12, when white letters were presented on a black background canvas, letter contours were first identified, creating a bounding box around them. These bounding boxes were then combined into a larger box enclosing the entire written letter. Ultimately, image preprocessing was applied to the resulting canvas image containing the letter within the larger box to ensure compatibility with the model’s expected input format.

##### Handling Colored Writing

**Figure 13:**
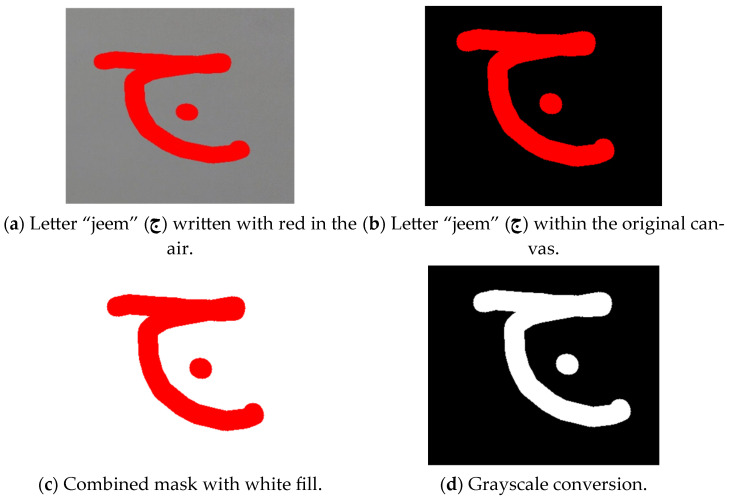
Image processing on the canvas.

For a canvas with colored writing, as illustrated in Figure 13, a distinctive approach was carried out. The colors taken individually were thresholded on their own to generate binary masks, which were then combined into a single mask representing the presence of any color. This combined mask was inverted to identify regions without writing, which were then filled with white color. The prescribed process resulted in a canvas with a white background and colored writing. To obtain a black background with white writing, the canvas was converted to grayscale by using an increased threshold value, and the colors of the grayscale image were inverted. In the end, adaptive thresholding was utilized for the inverted grayscale image to achieve a binary image featuring a black background and white writing.

In our study, the image processing work on the canvas encountered several challenges that significantly impacted the model’s recognition accuracy. One primary consideration was distinguishing between white writing and colored writing (red, green, or blue). For the white writing on a black background, the model’s expected input format was straightforward. However, processing colored writing required a different approach. Various image-processing techniques needed to be applied to the canvas to achieve the desired outcome of black-and-white writing.

When the writing was colored, a multi-step process was required to reach the final desired canvas. Initially, the canvas with colored writing needed to be transformed into a canvas with a white background and colored writing. This was accomplished by converting the image to grayscale, carefully selecting the grayscale intensity to ensure the writing appeared white. Determining the appropriate grayscale intensity was a challenge, as it needed to work effectively with all three colors (red, green, and blue).

**Figure 14:**
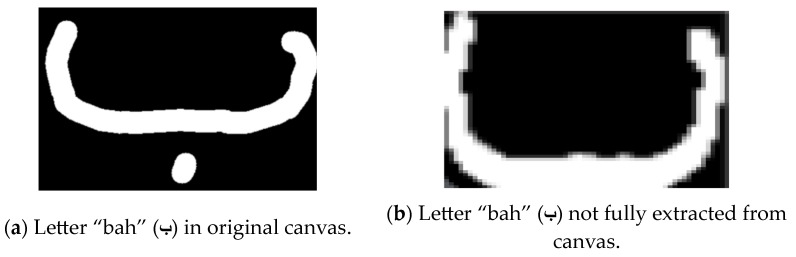
Initial cropping process.

**Figure 15:**
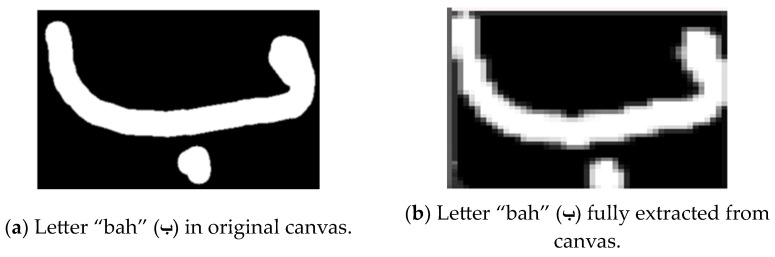
Modified cropping process.

Another key challenge was the complex structure of Arabic letters, which often consist of multiple parts like dots and hamza. As depicted in Figure 14, the initial cropping process did not include all of the necessary letter components, which problematically impacted the recognition results. To address this, as shown in Figure 15, a modified cropping process was implemented based on identifying the letter contours and enclosing them in a larger bounding box to represent the full letter.

#### 3.6.5. Model’s Recognition

Once the image has been processed, the handwritten Arabic letter is ready to be recognized by the optimal CNN architecture, which is the seven-layer CNN without dropout.

**Figure 16:**
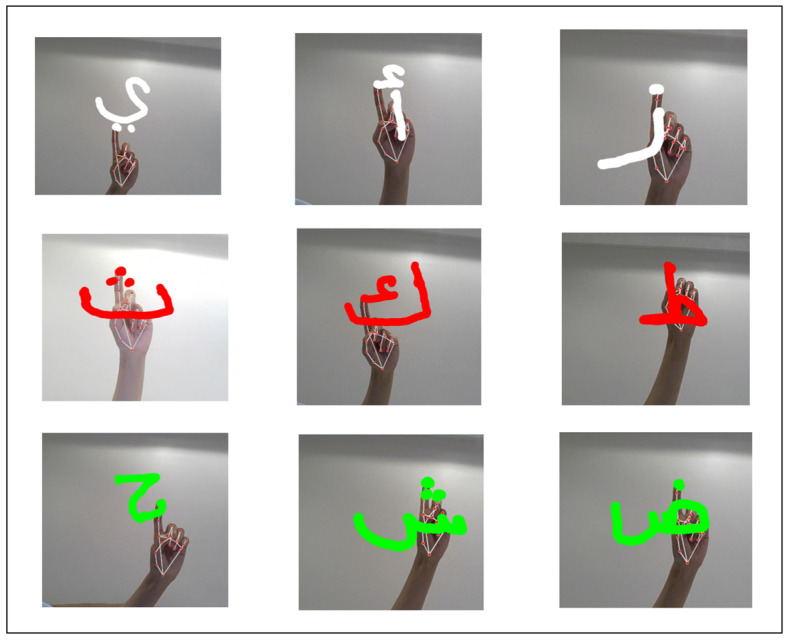
Samples of nine air-written Arabic letters.

Here are nine captured samples of air-written letters shown in Figure 16.

### 3.7. Frontend Development

**Figure 17:**
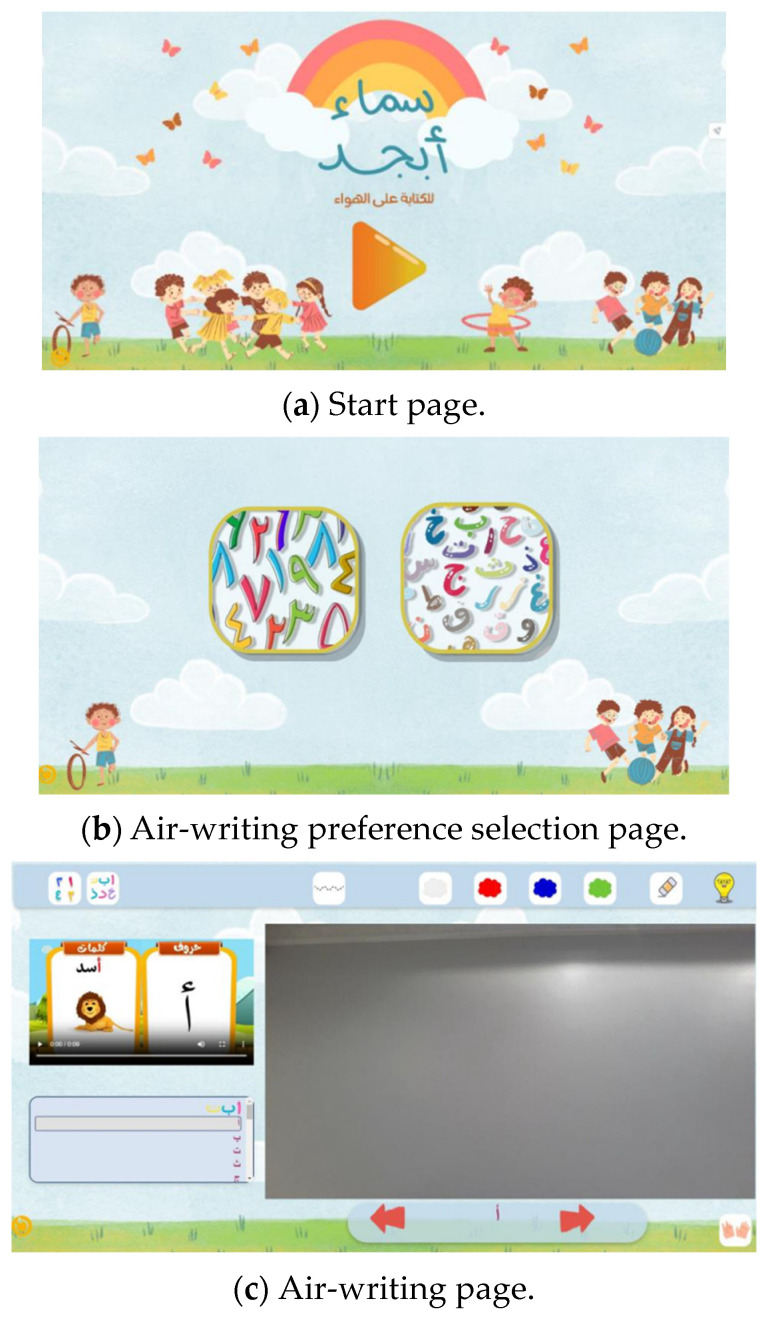
User interface of the SamAbjd web application.

The SamAbjd web application was thoughtfully designed to provide a remarkably user-friendly interface for the air-writing component, tailored specifically for children aged 5 to 7 years old. The application’s structure consists of three distinct HTML pages—a welcoming start page, a writing preference selection page, and the central air-writing page itself (Figure 17). These interactive pages were meticulously crafted using Hypertext Markup Language (HTML), Cascading Style Sheets (CSS), and JavaScript, all developed within the robust PyCharm coding environment.

Significantly, the SamAbjd web app incorporates voice-based instructions throughout and video tutorials of each letter [51,52], ensuring an intuitive and accessible experience for its young users. This thoughtful integration [53] of auditory guidance seamlessly complements the visually engaging interface, empowering even the youngest patrons to confidently explore and utilize the air-writing functionality.

## 4. Testing

Testing played a crucial role in the development of the SamAbjd web application, ensuring that it effectively met the needs of its target audience. Our testing approach included a detailed user testing phase and a satisfaction survey, both designed to assess the application’s usability, effectiveness, and overall user experience. These efforts provided valuable insights that guided the refinement of SamAbjd, making it a more user-friendly and efficient tool for children learning Arabic.

### 4.1. User Testing

The user testing phase of the SamAbjd application was a crucial step in evaluating the product’s usability, effectiveness, and overall user experience. This process involved real users, including children, mothers, and teachers, who interacted with the application to perform specific tasks. The testing aimed to identify any usability issues, gather valuable feedback, and assess how well the application meets the needs of its target audience.

A total of 15 participants were involved in user testing, consisting of 5 children, 5 mothers, and 5 teachers. These participants were selected based on the Nielsen Norman Group’s five-user rule, which states that “testing with 5 people lets you find almost as many usability problems as you’d find using many more test participants” [54]. The participants were asked to read and sign the consent form, which clarified that they participated voluntarily and that they could withdraw anytime. The consent form assured them that the collected data were anonymous and used for research purposes only. The guardians signed on behalf of their children who took part in this study. Each participant was assigned a series of tasks designed to mimic real-world usage of the SamAbjd application. The tasks included writing in the air using their index finger, pausing the writing, finishing the writing, changing the color of the writing, and clearing the writing. The participant’s performance was documented, focusing on completion time, the number of errors, and the number of attempts required to successfully complete each task.

In the first task, participants were required to lift their index finger and write letters or numbers in the air. Children took varying amounts of time to complete this task, ranging from 1 to 5 min, with some requiring multiple attempts. Despite the challenges, all participants successfully completed the task, demonstrating the application’s ability to recognize air-writing. The second task involved pausing the writing by lifting two fingers. This task proved more challenging, particularly for the children, with some taking up to 10 min to master the gesture. Teachers and mothers completed the task more quickly, with minimal errors, highlighting the need for improved gesture recognition for younger users.

To finish the writing, participants needed to open their full hands. Children again varied in their performance, with completion times ranging from 2 to 10 min. Teachers and mothers completed the task with ease, indicating that the gesture was generally intuitive but might require more practice for children.

Changing the color of the writing was a straightforward task that all participants completed quickly and without errors. The consistent success across all user groups suggests that this feature is well designed and easy to use.

Finally, participants were tasked with clearing the writing they had just completed. Like the previous task, this was performed quickly and without errors by all participants, confirming the effectiveness of the gesture.

The user testing results provided valuable insights into the performance and usability of the SamAbjd web application. While certain tasks posed challenges for younger users, particularly in terms of learning new gestures, the overall success rate was high. The feedback gathered from this testing phase will be instrumental in refining the application’s features and ensuring it meets the needs of its target audience effectively.

### 4.2. Real-Time Air-Writing Recognition Performance

The real-time performance from the end of the air-writing to recognition completion developed in this study reached milliseconds, which meets the target for usability, especially for applications targeting children. This high-quality real-time performance is a key strength of the proposed approach.

When comparing the real-time performance to related work, the researchers found that no prior papers had explored real-time air-writing recognition. To find a relevant benchmark, the team looked at a recent study [54] that introduced a real-time air-writing recognition system for Bengali characters using supervised machine learning. This prior work extensively evaluated the classification model by testing it 100 times on 10 randomly selected Bengali characters from their air-writing dataset.

Following the same evaluation of the performance methodology, the current study applied the trained classification model and achieved an accuracy of 84% in real-time recognition by using the following equation:

(4) Accuracy=CorrectAttempts×100

which is higher than the 81% accuracy reported in the related work. This improvement in real-time performance demonstrates the efficacy of the proposed approach. Table 3 summarizes the real-time performance results.

### 4.3. Satisfaction Survey

Following the testing phase, the same group of participants was surveyed to assess their satisfaction with the SamAbjd web application. The survey focused on the ease of learning gestures, the suitability of the interface for children, and the overall usability of the application.

Responses were measured on a scale from 1 to 5, with 1 indicating “strongly disagree” and 5 indicating “strongly agree”. The results, summarized in Table 4, show high levels of satisfaction across all user groups, particularly in terms of the application’s child-friendly design and intuitive gesture learning.

The feedback was overwhelmingly positive, with most users finding the application enjoyable, helpful, and suitable for children. These promising results demonstrate that the SamAbjd application has been well received and is effective in its purpose. The positive responses also provide motivation to continue improving and enhancing the application, ensuring it remains an advanced and engaging tool for children to learn and practice Arabic.

## 5. Discussion and Comparison with Existing Work

With a shared focus on air-writing recognition, related papers provide valuable insights and analysis, albeit through different lenses and methodologies. We are going to compare our study with a paper titled “Recognition of Arabic Air-Written Letters: Machine Learning, Convolutional Neural Networks, and OCR Techniques” by Nahar et al. [30], which explores the recognition of Arabic letters written in the air.

Our paper takes a different approach to air-writing recognition. The motivation behind our study is to enhance children’s cognitive development by teaching them to write the Arabic alphabet. We observed a lack of Arabic language educational applications, which led us to develop an interactive web application called SamAbjd. This application leverages deep learning techniques, including air-writing recognition, to facilitate the learning of Arabic letters.

S. Nahar et al. [30] used one dataset, and they gathered from it 9000 images of Arabic alphabets, focusing only on 18 letters in positions within words, such as the beginning, middle, or end.

Regarding data preprocessing, both papers applied image denoising. However, in our study, we additionally used data augmentation and grayscale conversion, while Nahar et al. [30] employed feature extraction, dimensionality reduction, and data normalization.

In terms of methodology, we used deep learning models, specifically CNNs and VGG16. We conducted a series of adjustments on the training parameters to evaluate their effectiveness in recognizing handwritten Arabic letters. On the other hand, Nahar et al. [30] used a hybrid model combining feature extraction, deep learning models, machine learning methods, and OCR techniques. They applied various machine learning algorithms, including neural networks (NNs), random forest (RF), KNN, and support vector machine (SVM). Deep features extracted from CNNs such as VGG16, VGG19, and SqueezeNet were fed into the machine learning models.

In terms of results, we achieved a high accuracy of 96.40% using a seven-layer CNN model without dropout. In comparison, Nahar et al. [30] achieved an accuracy of 88.8% using an NN with VGG16.

Furthermore, our study includes a practical implementation of the proposed system, an actual web application with a frontend interface, while Nahar et al. [30] did not mention the existence of an application.

Our approach stands out due to several key factors that collectively contribute to its superior performance. Firstly, we focused primarily on deep learning, utilizing CNNs. This deliberate choice was driven by the remarkable results achieved using CNNs. They offered superior performance, faster processing speeds, reduced computational load, and minimized complexity compared to other approaches. Moreover, the size of our dataset played a pivotal role in enhancing our approach. By utilizing a larger number of images, we were able to significantly improve the accuracy of our model.

Table 5 highlights the key differences between our study and the work conducted by Nahar et al. [30].

Additionally, we devoted considerable effort to studying and implementing effective preprocessing techniques for our dataset. One notable technique involved converting all images to grayscale, which proved beneficial in aiding the training process of our model. Furthermore, we included data augmentation techniques, such as rotating, zooming, and shifting the Arabic letter images. This ensured that a wide range of variations were generated in the dataset. We believe this data augmentation approach is a key advantage of our study compared to prior work, as the increased dataset diversity will be particularly beneficial for recognizing the air-writing of the target audience—young children using the SamAbjd air-writing web application.

## 6. Conclusions and Future Work

The study was developed to teach children how to write Arabic letters in a novel and captivating way using deep learning techniques, specifically air-writing recognition. The data for this study were sourced from three publicly available datasets, yielding a substantial collection of 31,349 annotated images of handwritten Arabic letters. To enhance the dataset quality, various preprocessing techniques were implemented, including image denoising, grayscale conversion, and data augmentation. These steps were essential for preparing the data for training the recognition model.

Two models, a CNN and VGG16, were evaluated for their performance in recognizing handwritten Arabic characters. Among the CNN models tested, a seven-layer model without dropout demonstrated superior performance, achieving high training and testing accuracies of 95.92% and 96.40%, respectively. This indicates a successful fit of the model without overfitting. The model also achieved high precision and an F1-score of 96.43%.

To provide a user-friendly interface, a web application was developed in association with the recognition model using Flask and PyCharm. This development ensures a robust and efficient platform for children to engage with and learn the Arabic alphabet and numbers.

A limitation of our current work is the camera’s performance, as we aim for it to function consistently across various weather conditions and different devices.

Future work will focus on expanding the system’s capabilities by incorporating a number recognition model and developing a mobile application. Additionally, we plan to create a plugin that can be integrated into educational platforms, significantly benefiting online learning. We also plan to experiment with more advanced architectures and hybrid models to enhance the accuracy of capturing air-writing.

## Figures and Tables

**Table 1:** Hyperparameters for the selected models.

Epoch	Batch Size	Learning Rate
50	0.001	400
20	0.001	515
25	0.02	515
20	0.001	512

**Table 2:** Results of the conducted experiments on CNN and VGG16 models.

Model	Epoch	Learning Rate	Batch Size	Train Loss (%)	Train Acc. (%)	Valid Loss (%)	Valid Acc. (%)	Test Acc. (%)	Precision (%)	F1-Score (%)
CNN 7 layers without dropout	**50**	**0.001**	**400**	**3.53**	**98.85**	**11.35**	**97.03**	**97.77**	**97.83**	**97.82**
	20	0.001	515	12.46	95.92	16.46	94.70	96.40	96.44	96.43
	25	0.02	515	9.81	96.77	14.78	95.37	96.65	96.78	96.72
	20	0.001	512	12.13	96.04	18.31	94.00	96.36	96.47	96.41
CNN 7 layers with dropout	50	0.001	400	22.04	92.85	20.40	93.59	95.73	95.83	95.80
	20	0.001	515	34.87	89.10	23.59	92.03	94.41	94.50	94.44
	25	0.02	515	26.34	91.69	18.15	93.97	95.98	96.09	96.07
	**20**	**0.001**	**512**	**28.63**	**91.11**	**20.41**	**94.13**	**95.94**	**96.10**	**96.05**
CNN 10 layers without dropout	**50**	**0.001**	**400**	**2.74**	**99.07**	**8.60**	**97.67**	**98.12**	**98.23**	**98.20**
	20	0.001	515	6.09	97.97	11.65	96.81	97.70	97.59	97.73
	25	0.02	515	5.06	98.34	11.57	96.52	97.54	97.59	97.58
	20	0.001	512	15.02	95.33	18.49	94.86	96.97	97.12	97.04
VGG16 with fully connected layer	50	0.001	400	3.84	72.23	11.76	71.87	70.62	71.32	69.10
	20	0.001	515	2.76	69.46	12.98	69.77	71.83	70.56	70.18
	25	0.02	515	4.98	71.73	10.73	70.63	72.53	72.98	72.65
	**20**	**0.001**	**512**	**2.32**	**73.54**	**9.43**	**72.39**	**72.82**	**71.74**	**72.70**
VGG16 without fully connected layer	**50**	**0.001**	**400**	**61.98**	**79.08**	**80.23**	**75.84**	**76.24**	**74.02**	**75.02**
	20	0.001	515	95.81	72.75	106.91	70.44	71.17	69.63	70.07
	25	0.02	515	72.62	80.60	149.41	75.4	75.08	73.80	73.89
	20	0.001	512	72.56	77.00	88.23	74.24	74.28	72.19	73.06

**Table 3:** Real-time performance of the model in recognition of Arabic air-writing letters.

Character	No. of Attempts *										Accuracy
	1st	2nd	3rd	4th	5th	6th	7th	8th	9th	10th	
(أ) Alef	ع	ع	ع	ع	ع	ع	✔	✔	ع	ع	20%
(ف) Feh	✔	✔	✔	✔	✔	✔	✔	✔	✔	✔	100%
(ي) Yeh	✔	✔	✔	✔	✔	✔	✔	✔	✔	✔	100%
(ك) Kaf	✔	✔	✔	✔	✔	✔	✔	✔	✔	✔	100%
(ق) Qaf	✔	✔	✔	✔	✔	✔	✔	✔	✔	✔	100%
(ب) Beh	✔	✔	ت	ت	✔	ت	✔	ت	✔	✔	60%
(ز) Zeh	✔	✔	✔	✔	✔	✔	✔	✔	✔	✔	100%
(د) Dal	✔	✔	✔	✔	✔	✔	✔	✔	✔	✔	100%
(ص) Sad	✔	✔	✔	س	✔	✔	✔	س	س	✔	70%
(ذ) Thal	✔	✔	✔	✔	✔	ز	✔	✔	✔	✔	90%
**Overall Accuracy**											84%

* The ✔ symbol indicates a correct prediction, while a letter represents the incorrect prediction made by the model.

**Table 4:** Results of the satisfaction survey responses.

User Type	Is the Web Application Easy for the Child to Use?	Are the Colors and Interfaces Suitable for Children?	Was It Easy to Learn and Perform the Gestures in the Air?	Average
Children	5	5	4	13.4
	4	5	3	
	4	5	5	
	3	5	4	
	5	5	5	
Mothers	5	5	4	14.2
	5	5	5	
	4	5	4	
	5	5	5	
	5	5	4	
Teachers	5	5	5	14.6
	5	5	5	
	5	4	5	
	5	5	5	
	5	5	4	

**Table 5:** Summary of comparison between our study and Nahar et al.’s study [30].

	Our Study	Nahar et al. [30]
**Motivation**	The motivation behind our study is to enhance children’s cognitive development by teaching them to write the Arabic alphabet. We observed a lack of Arabic language educational applications.	This study aimed to develop and evaluate an air-writing recognition system for the Arabic language, addressing the lack of research in this area compared to English and Chinese.
**Dataset**	We gathered data from three different sources, resulting in 31,349 annotated images of all **28** Arabic letters.	The study utilized the AHAWP dataset, a comprehensive collection of letters, words, and paragraphs written in Arabic, containing **18** letters. The dataset was gathered from 82 individuals, comprising a total of 9000 images of the Arabic alphabet in various positions within words.
**Data Preprocessing**	Image denoising, data augmentation, and grayscale conversion.	Feature extraction, dimensionality reduction, data normalization, and image denoising.
**Methodology**	Deep learning	Deep learning and machine learning
**Models**	CNN and VGG16	Machine learning: NNs, RF, KNN, SVM.
		Deep learning: CNN, VGG16, VGG19, and SqueezeNet.
**Best Model**	7-layer CNN model without dropout	NN with VGG16.
**Accuracy**	96.40%	88.80%
**Practical Implementation**	Our study includes a practical implementation of the proposed system, an actual web application with a frontend interface.	
		Did not mention the existence of an application.

## Data Availability

Data are contained within the article.
